# Surface-Enhanced Raman Scattering from Dye Molecules in Silicon Nanowire Structures Decorated by Gold Nanoparticles

**DOI:** 10.3390/ijms23052590

**Published:** 2022-02-26

**Authors:** Saltanat B. Ikramova, Zhandos N. Utegulov, Kadyrjan K. Dikhanbayev, Abduzhappar E. Gaipov, Renata R. Nemkayeva, Valery G. Yakunin, Vladimir P. Savinov, Victor Yu Timoshenko

**Affiliations:** 1 Faculty of Physics and Technology, Al-Farabi Kazakh National University, 71, Almaty 050040, Kazakhstan; ykramova.saltanat@kaznu.kz (S.B.I.); dksolar2017@gmail.com (K.K.D.); 2 Department of Physics, School of Sciences and Humanities, Nazarbayev University, Nur-Sultan 010000, Kazakhstan; 3 Department of Medicine, Nazarbayev University School of Medicine, Nur-Sultan 010000, Kazakhstan; abduzhappar.gaipov@nu.edu.kz; 4 National Nanotechnology Laboratory Open Type, Faculty of Physics and Technology, Al-Farabi Kazakh National University, Almaty 050040, Kazakhstan; quasisensus@mail.ru; 5 Faculty of Physics, Lomonosov Moscow State University, 119991 Moscow, Russia; yvg51@bk.ru (V.G.Y.); savinov1983@yahoo.com (V.P.S.); 6 Lebedev Physical Institute of the Russian Academy of Sciences, 119991 Moscow, Russia

**Keywords:** surface-enhanced Raman scattering, plasmonics, gold, nanoparticles, silicon, nanostructures, mesopores, nanowires, molecular sensorics

## Abstract

Silicon nanowires (SiNWs) prepared by metal-assisted chemical etching of crystalline silicon wafers followed by deposition of plasmonic gold (Au) nanoparticles (NPs) were explored as templates for surface-enhanced Raman scattering (SERS) from probe molecules of Methylene blue and Rhodamine B. The filling factor by pores (porosity) of SiNW arrays was found to control the SERS efficiency, and the maximal enhancement was observed for the samples with porosity of 55%, which corresponded to dense arrays of SiNWs. The obtained results are discussed in terms of the electromagnetic enhancement of SERS related to the localized surface plasmon resonances in Au-NPs on SiNW’s surfaces accompanied with light scattering in the SiNW arrays. The observed SERS effect combined with the high stability of Au-NPs, scalability, and relatively simple preparation method are promising for the application of SiNW:Au-NP hybrid nanostructures as templates in molecular sensorics.

## 1. Introduction

The phenomenon of surface-enhanced Raman scattering (SERS) was first observed on a rough silver electrode with a pyridine monolayer in 1974 [1], and it has been studied for years (see, for example, Refs. [2,3]). Nowadays, the SERS effect is widely used for detecting target molecules at low concentrations that are important for numerous applications, from ecology [4] and chemistry [5] to biosensorics [6,7,8], including biomedical diagnostics [9,10,11,12]. The most convenient explanation of SERS considers two main mechanisms, i.e., (i) electromagnetic enhancement and (ii) chemical one [3]. While the contribution of the latter is usually considered much smaller than electromagnetic enhancement [3], both mechanisms are sensitive to the physical properties and morphology of SERS substrates [5,6]. Now, the most prominent SERS-active substrates employ an effect of the enhanced electric field due to localized surface plasmon resonance (LSPR) in what are called plasmonic metal nanoparticles (NPs) [3,4]. Silver (Ag) and gold (Au) NPs with amazing plasmonic characteristics are usually used to detect low concentrations of biomolecules by SERS [13,14,15,16,17]. While the SERS signal promoted by individual plasmonic NPs is characterized by relatively low intensity and poor reproducibility, which become a major obstacle for practical application and commercialization, the use of specifically designed nanostructured substrates as templates for plasmonic NPs is the main direction for the development of SERS sensing systems for different biomolecules [16], living cells [17], and biosystems [18,19,20].

In recent years, various types of semiconductor nanostructures such as silicon nanowires (SiNWs), nanotubes, and porous films have attracted research interest as templates for SERS-active plasmonic NPs because of the high surface-to-volume ratio, roughness, and the tunability of pore sizes in the former [19,20,21,22,23]. For example, SiNWs [22] and amorphous/crystalline hybrid Si-based nanostructures [23] have opened a broad avenue for developing new SERS substrates, which complement pure metallic nanostructures [2,3,6]. Also, electrochemically prepared porous silicon (por-Si) and por-Si based 1D photonic crystals with deposited Ag-NPs have been explored as potential SERS templates with a detection limit up to 10^−12^ M [24]. Prior to this, SiNWs with deposited Ag-NPs were used for ultrasensitive detection of biomolecules [25].

Besides SERS application, SiNWs are extensively studied for their use in photovoltaics [26], photocatalysis [27], gas sensors [28], drug delivery [29], and biomedical diagnostics [30]. Modern methods for the preparation of SiNWs consist of different physical and chemical approaches, i.e., reactive ion etching [31], thermal evaporation [25,32], laser ablation [33], vapor–liquid–solid growth [34], plasma etching [35], molecular beam epitaxy [36], and metal-assisted chemical etching (MACE) [37,38]. Among them, MACE is very suitable because of the simplicity, low cost, and possibilities for tailoring the morphology of SiNW arrays, e.g., their surface roughness, length, porosity, and spacing between individual Si nanostructures [36,37]. Features of the spatial distribution of catalytic metal NPs on the surface of c-Si substrate, such as uniformity, density, and thickness, will affect the morphology of Si nanostructures formed during MACE [37]. When NPs of silver and gold as catalysts are deposited onto the c-Si surface, the MACE-formed structures can be changed from mesoporous Si layers to arrays of SiNWs with an increase in the spatial density of metallic NPs [36,37,38].

As for SERS application, SiNW arrays are usually decorated with Ag or Au-NPs by using chemical methods [39,40,41,42]. The advantages of chemical deposition include its implementation and simplicity; self-induced deposition does not need vacuum technology or a power supply [41]. Ag-NPs usually exhibit better plasmonic enhancement of SERS, but they do not possess surface stability and biocompatibility in comparison with Au-NPs [42]. Moreover, by adjusting the size, shape, and concentration of Au-NPs on the surfaces of semiconductor-based nanostructures, one can create new photonic materials with unique properties for biosensing applications [8,43,44,45,46].

The performance of SERS-active surfaces is usually characterized by an enhancement factor, which depends on the substrate morphology and physical properties of plasmonic NPs [2,3]. Maximal SERS efficiency is often associated with the formation of a so-called “hot spot” of the electric field related to plasmonic NPs [3,17,18]. The reproducibility and stability of the SERS signal is an important metric for evaluating the effectiveness of SERS-active surfaces, which means that the surface must be well controllable and stable for a long time [45,46,47]. So, the search for a versatile and high-performance substrate for the SERS of biomolecules is still ongoing, with well-known technologies being developed and new fabrication technologies being proposed.

Here, we investigate Au-NP-decorated SiNWs prepared using the MACE method followed by deposition of additional Au-NPs. The prepared samples, which represent either highly porous Si layers or arrays of relatively well spatially separated bundles of SiNWs, are tested as templates for the SERS detection of dye molecules of methylene blue (MB) and Rhodamine B (RB) with molar concentration varying from 10^−15^ to 10^−6^ M. The obtained results allow us to reveal an effect of the morphology of SiNWs on their activity as potential SERS substrates for the highly sensitive detection of molecules.

## 2. Results and Discussion

**Figure 1:**
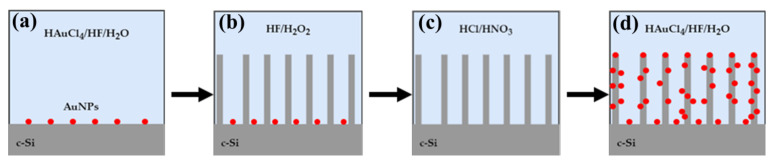
Schematic illustration of the fabrication process of SiNWs:Au-NPs: (**a**) deposition of catalytic Au-NPs on c- Si surface in HAuCl_4_/HF/H_2_O; (**b**) Au-NP-assisted etching with the HF/H_2_O_2_ solution; (**c**) removal of residual Au-NPs in HCl/HNO_3_ solution; (**d**) deposition of Au-NPs from HAuCl_4_/HF/H_2_O into SiNW arrays.

Figure 1 shows a schematic view of the preparation procedure of SiNWs:Au-NPs. The whole process consisted of four stages. In the first stage (Figure 1a), c-Si substrates were immersed in an aqueous solution of HAuCl_4_/HF to deposit Au-NPs, which were used to catalyze the heterogeneous dissolution of c-Si in hydrofluoric acid, i.e., MACE [42]. Three main series of the samples were prepared with Au-NP deposition times, *t_dep_*, which were 10, 20, and 30 s. The deposition of Au-NPs was carried out by immersing the c-Si substrate into an aqueous solution of HAuCl_4_ (0.4 mM)/HF (5 M) for *t_dep_*. Different deposition times of Au-NPs were used to prepare series of samples with different surface densities of pores, i.e., low, medium, and high porosity. The resulting samples were washed with deionized water and dried in air. Then, c-Si wafers with deposited Au-NPs were etched in a solution of HF/H_2_O_2_ by using the MACE process (Figure 1b). The etching solution for MACE was a mixture of an aqueous solution consisting of HF (5 M, 40%) and H_2_O_2_ (37%) with a volume ratio of 2:10; the etching time was 30 min. Then, the samples were immersed in a mixture of HCl/HNO_3_ = 3:1 to remove residual Au-NPs (Figure 1c). Finally, the samples were immersed into the HAuCl_4_/HF solution to deposit Au-NPs on the SiNW’s surfaces (Figure 1d).

**Figure 2:**
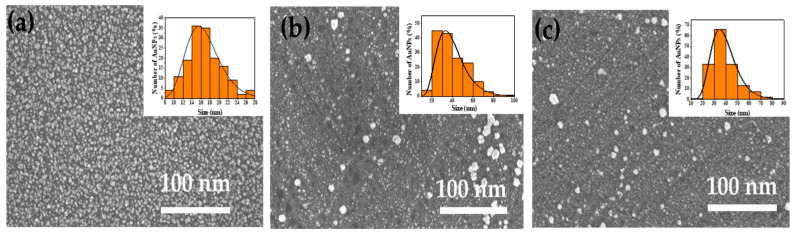
SEM images of Au-NPs deposited on the surface of c-Si wafers for different deposition times: (**a**) 10 s, (**b**) 20 s, and (**c**) 30 s. The corresponding size of distribution for Au-NPs (orange bars) and their fits by log-normal functions (solid line) are shown in the insets.

Figure 2 shows typical top-view SEM images of Au-NPs deposited on the surface of c-Si wafers for different deposition times. Au-NPs are randomly distributed on the surface, and the size distribution can be described by the log-normal functions (insets of Figure 2) with mean sizes of about 16, 32, and 35 nm for the deposition times of 10, 20, and 30 s, respectively. These samples with different size distributions of Au-NPs were proceeded by MACE to form SiNW arrays with different morphologies.

**Figure 3:**
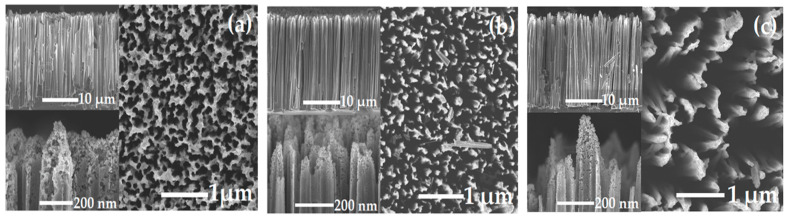
Top-view SEM images of SiNWs:Au-NPs samples prepared at the following time *t_dep_*: (**a**) 10 s, (**b**) 20 s, and (**c**) 30 s. Upper and lower insets show lateral views of whole arrays and tips of SiNWs, respectively.

Figure 3 shows typical top-view SEM images of SiNWs:Au-NP structures and upper insets show the lateral view of the corresponding samples. The total thickness of the SiNWs layer is about 34–35 µm for all samples, since it is controlled by the MACE duration. However, the samples prepared with different *t_dep_* consist of SiNW arrays with different morphologies, and the mean spacing between neighboring NWs is controlled by the size distribution of catalyzing Au-NPs deposited at the first preparation stage (Figure 1a).

To quantify the volume fraction of pores, which are etched in the c-Si substrate due to the MACE process by catalytic Au-NP, we used an analysis by the box-counting method [48] of top-view SEM images of the prepared samples (Figure 3). According to this analysis (Supplemental Figure S1 and Equation (S1)), the porosity, *P*, accounts for 55, 72, and 83% for *t_dep_* = 10, 20, and 30 s, respectively. One can see that the sample with *P* = 55% can be considered a porous layer with partially fused mesopores, which form a dense array of SiNWs (Figure 3a). However, the sample with *P* = 72% looks like an array of relatively well-defined SiNWs with mean cross-sectional sizes of the order of 100 nm (Figure 3b). Furthermore, the sample with *P* = 83% represents an array of SiNWs with significantly larger spacing between nanowires (Figure 3c).

The down insets of Figure 3 show SEM images of SiNW’s tips with deposited plasmonic Au-NPs, which are seen as small black spots. The EDX analysis (Supplemental Figures S2–S4) indicates that the total concentration of gold is 3.2, 3.8, and 3.0 weight % for the samples with *P* = 55, 72, and 83%, respectively. Thus, the gold concentration is about 0.45 ± 0.05 at. %, and it is weakly dependent on the sample porosity.

**Figure 4:**
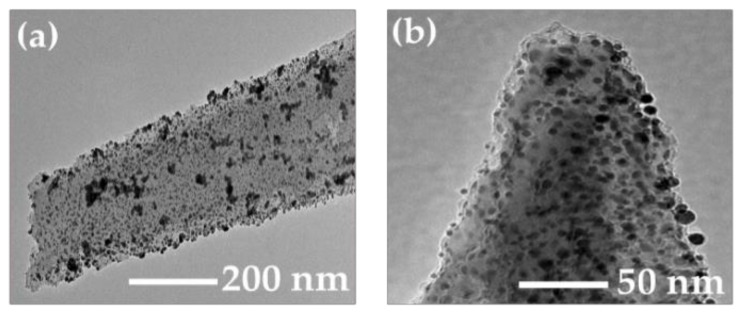
TEM images of (**a**) individual SiNW with deposited Au-NPs and (**b**) tip of such nanowire.

Figure 4 shows TEM images of an individual SiNW with deposited Au-NPs. The mean size of Au-NPs is about 10 nm, as shown in Figure 4b. This value is close to the size of Au-NPs, which is determined by the same deposition time for the flat surfaces of c-Si wafers, as shown in Figure 2. Besides isolated Au-NPs, there are many of their aggregates attached to the side surfaces (Figure 4a) and tips (Figure 4b) of SiNWs. The surface roughness of the latter promotes the tight binding between Au-NPs and SiNWs. 

**Figure 5:**
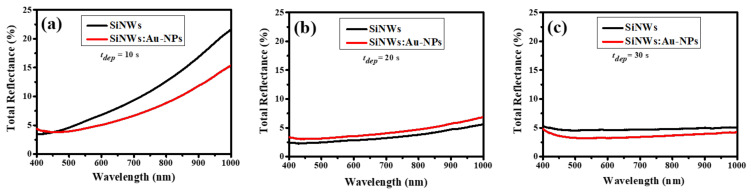
Spectra of the total reflectance for SiNWs and SiNWs:Au-NPs with different *t_dep_* (**a**) 10 s; (**b**) 20 s; (**c**) 30 s, respectively.

Figure 5a shows that the total reflection for SiNWs and SiNWs:Au-NP samples increases from about 4 to 26% in the spectral range from 400 to 1000 nm, while the samples with *t_dep_* = 30 s exhibit significantly lower reflectance of about 6–11% (Figure 5c). In the case of Au-NPs deposited on the c-Si surface for 20 s, the total reflection for SiNWs:Au-NPs shows higher reflectivity than that for pure SiNW arrays of the same porosity (Figure 5b). The low reflectivity of SiNW arrays is usually related to an effect of light localization, which is caused by strong elastic light scattering accompanied with light absorption in Si nanostructures [49]. The porosity of 55% for SiNW arrays likely promotes weaker light localization, which seems to be a little stronger for the sample of SiNWs:Au-NPs, as evidenced by the lower reflectance coefficient of the latter (Figure 5a).

**Figure 6:**
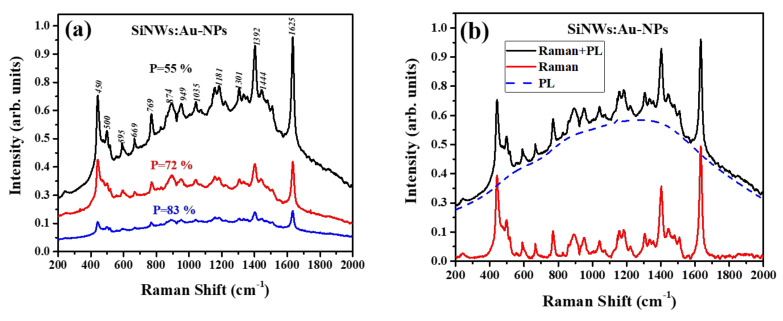
(**a**) Raman and PL spectra measured for SiNWs:Au-NPs arrays with porosity *P* = 55% (black line), 72% (red line), and 83% (blue line) after deposition of MB molecules (10^−6^ M); (**b**) the same spectrum for the sample with porosity 55% (black line) and its deconvolution by SERS (red line) and PL (dashed blue line) spectra.

Figure 6a shows spectra of SiNWs:Au-NPs with adsorbed MB molecules. The spectra consist of narrow peaks of the SERS from MB molecules together with a broad background repeated to the photoluminescence (PL) of MB. The intensities of both SERS and PL decrease with increasing sample porosity. At the same time, the Raman intensity of MB was very weak for initial SiNWs without Au-NPs. The increase in the Raman signal for SiNWs with deposited Au-NPs in comparison with the initial ones is obviously explained by the SERS effect because of the LSPR-enhanced electric fields nearby Au-NPs. The difference in the SERS enhancement for SiNW arrays with different porosity cannot be explained by different concentrations of Au-NPs, which are approximately the same (0.4–0.5 at.%) for all samples of SiNWs:Au-NPs (Supplemental Figures S2–S4 and Table S1).

To reveal the net Raman signal in SiNWs:Au-NPs structures, the PL background is subtracted from the total signal, as shown in Figure 6b. The PL spectrum is interpolated by a polynomial function centered near 1300 cm^−1^, which corresponds to a wavelength of 690 nm, in agreement with the well-known fluorescence spectrum of MB [50]. The spectrum with the subtracted PL contribution allows us to evaluate the net SERS effect in SiNWs:Au-NPs.

**Figure 7:**
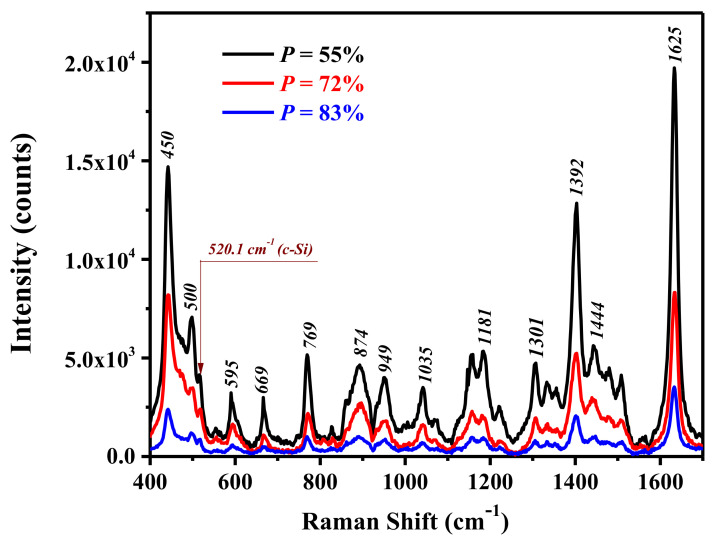
SERS spectra of SiNWs:Au-NPs samples with different porosity *P* after adsorption of 10^−6^ M of MB molecules.

Figure 7 shows that besides the Raman line of MB molecules, a line at 520.5 cm^−1^ is observed in the spectra of all investigated samples. This line is obviously related to the one-phonon Raman scattering by the crystalline lattice of SiNWs [49]. While the intensity of this line increases with decreasing total porosity of the samples, the SERS signal of MB increases even more and reaches a maximal value for the sample with *P* = 55% (black curve in Figure 7).

The SERS spectra of MB consist of numerous peaks with frequencies at 450, 500, 595, 669, 769, 874, 949, 1035, 1181, 1301, 1392, 1444, and 1625 cm^−1^, which correspond to different vibration modes of MB molecules [47]. The assignment of the Raman active vibration modes of MB is given in Table 1.

**Figure 8:**
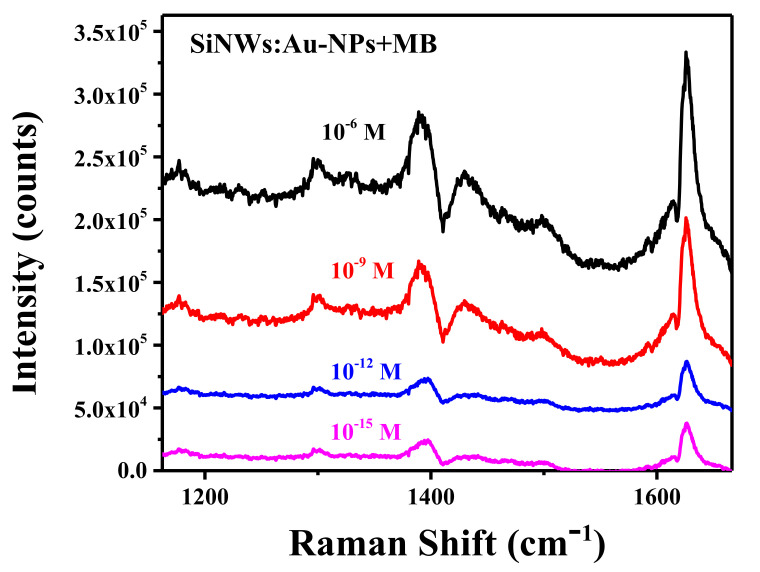
SERS spectra of SiNWs:Au-NPs (*P* = 55%) with different concentrations of deposited MB molecules.

Figure 8 shows SERS spectra of MB adsorbed with different concentrations on the surfaces of SiNWs:Au-NPs. One can see that the MB concentration of the order of 10^−15^ M can be easily detected for the samples with *P* = 55%, indicating the high efficiency of the prepared SERS substrates. As for the MB concentration dependence of the SERS intensity, it is weakly sensitive to the molecule concentration in the range from pico- to femtomolar. This fact can be related to the certain amount of MB molecules adsorbed in the hottest spots formed by neighboring Au-NPs on the surfaces of SiNWs, similar to the case of dried colloidal Au-NPs drop casted on a glass slide [47]. It should be noted that in our experiments, the SERS signal of MB adsorbed on flat c-Si substrates coved with Au-NPs deposited for 10 s was well detectable only at a rather high concentration of about 1 µM (Supplemental Figure S6) and it was nonreproducible at lower MB concentrations. The maximal SERS signal of MB adsorbed on the c-Si wafer with deposited Au-NPs was at least an order of magnitude lower than that for SiNWs:Au-NPs. This fact demonstrates that the latter structures promote the SERS efficiency due to the high surface-to-volume ratio of Si nanowires combined with the electromagnetic enhancement of the Raman scattering.

We carried out additional experiments on the SERS efficiency of SiNWs:Au-NPs where we increased the incubation time of Au-NPs from 10 to 80 s at the last stage of preparation (Figure 1d). The experimental results showed that the SERS signal depended nonmonotonically on the incubation time and that it could be possible to additionally improve the SERS signal by about 1.5–2 times by using an incubation time of 80 s. However, the SERS signal improvement was accompanied with an increase of the PL background (Supplemental Figure S7). For shorter and longer incubation times, the SERS activity decreases. The nonmonotonic dependence of the SERS efficiency on the incubation time can be explained by an interplay between the creation of additional hot spots of the electric field nearby Au-NPs and the light absorption related to stronger light localization in arrays of SiNWs with deposited Au-NPs. Moreover, the SERS activity of SiNWs:Au-NPs samples prepared for longer incubation times can be influenced by aggregation of Au-NPs that will change both their mean size and morphology.

To quantify the SERS efficiency of the investigated samples, we analyzed an enhancement factor (*EF*), which can be expressed by the following equation [3]:

(1) $$EF=\frac{I_{S}\cdot C_{ref}}{C_{S}\cdot I_{ref}}$$

where *I_S_* and *I_ref_* are the Raman intensities of MB for the analyzed sample, i.e., SiNWs:Au-NPs, and reference sample, respectively; *C_S_* and *C_ref_* are the MB concentrations in the analyzed and reference samples, respectively. If initial SiNWs are used as the reference sample, it allows us to assume the similar surface distribution of MB molecules in the samples.

Using Equation (1), the *EF* factor for the Raman line of MB at 1625 cm^−1^ is estimated to be 6.1 × 10^4^, 2.6 × 10^4^, and 1.1 × 10^4^ for the samples with *P* = 55, 72, and 83%, respectively (for details, see Figure S5). The maximal *EF* for SiNWs:Au-NPs with lower porosity can be related to the optimal morphology SiNW arrays, which control partial light localization in a combination with LSPR-enhanced electric fields nearby Au-NPs. The latter effect can also be stronger for rough surfaces at the nanoscale [14]. 

Table 1 summarizes the *EF* values of SiNWs:Au-NPs to MB with a concentration of 10^−6^ M for different molecular vibration frequencies. One can see that the strongest *EF* occurs for the band at 1444 cm^−1^, which can be assigned to the mixed vibrations coupled with ν(C―C)/ν(C―N) stretching and in plane *β*(CH) bending vibrations of the fused aromatic ring of the MB molecule [47]. The maximal *EF* for these vibration modes as well the *EF* variations for different bands can be related to an effect of the chemical factor of SERS [3], which influences the Raman polarizability of adsorbed dye molecules. 

**Figure 9:**
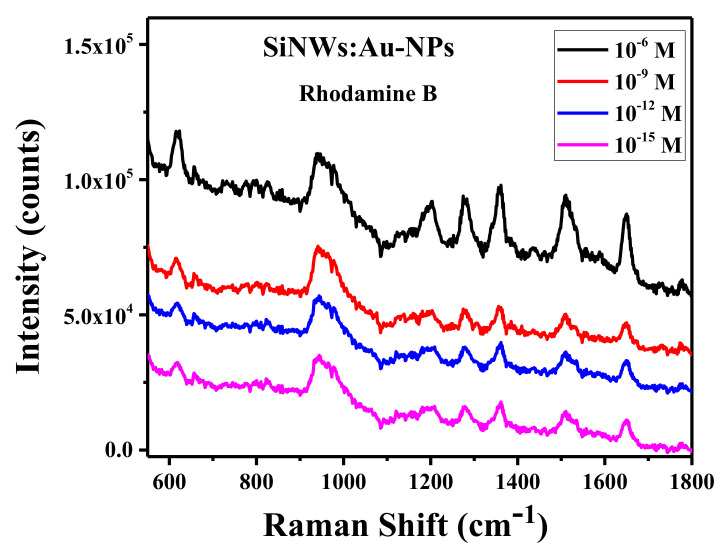
SERS spectra of SiNWs:Au-NPs with *P = 55*% after deposition of Rhodamine B with different molar concentrations.

To further demonstrate the high sensitivity of our sensory platform, the SERS spectra of RB molecules were measured in the range of molecule concentration from 10^−15^ to 10^−6^ M (Figure 9). Similar to the case of MB, the SERS signal of RB was weakly dependent on the molecule concentrations varying from 10^−6^ to 10^−9^ M. This fact can indicate both high sorption efficiency and tight binding of RB molecules at the hot spots between Au-NPs on the SiNW’s surfaces. Such hot spots should be characterized by enhanced strength of the electric field, which will additionally bind and polarize the dye molecule [3,47,51]. The same effect is probably responsible for the *EF* spreading, which is observed for MB molecules, as shown in Table 1. 

## 3. Materials and Methods

### 3.1. Chemistry and Materials

Arrays of SiNWs were formed on optically polished 350 μm-thick (100)-oriented c-Si wafers of p-type conductivity (boron-doped, specific resistivity of 1–10 Ohm×cm). All chemicals, i.e., Chloroauric acid (HAuCl_4_·xH_2_O, CAS No. 27988-77-8), Methylene blue (C_16_H_18_ClN_3_S·xH_2_O, CAS No. 122965-43-9), Rhodamine B (C_28_H_31_ClN_2_O_3_, CAS No. 81-88-9), Hydrofluoric acid (HF, 40%, CAS No. 7664-39-3), Hydrogen peroxide (H_2_O_2_, 30%, CAS No. 7722-84-1), Acetone (CH_3_COCH_3_, 99.9%, CAS No. 67-64-1), Hydrochloric acid (HCl, 37%, CAS No. 7647-01-0), and Sulfuric acid (H_2_SO_4_, 95–98%, CAS No. 7664-93-9) were purchased from Sigma-Aldrich.

### 3.2. MACE Formation of SiNWs

Samples were fabricated by standard two-step MACE by using chemically deposited Au-NPs as catalysts [36]. First, optically polished c-Si wafers were ultrasonically cleaned for 7 min in acetone to remove surface organic contamination. Then, they were rinsed with deionized water and subsequently cleaned with a piranha solution (containing 98% H_2_SO_4_ and 37% H_2_O_2_ with a volume ratio of 3:1) for 10 min, then washed in deionized water. The Si wafers were then rinsed with 5% HF for 3 min to remove the natural SiO_2_ layer on the surface of the samples and finally bathed again with deionized water.

### 3.3. Preparation of SiNW:Au-NP Structures

Plasmonic Au-NPs were deposited on the surfaces of SiNWs in a solution of HAuCl_4_ (0.4 mM)/HF (5 M) for 10 s. Then, the obtained SiNWs:Au-NP structures were washed with deionized water and dried in air. All manufacturing processes were carried out at room temperature.

### 3.4. Adsorption of Dye Molecules on SERS Substrates

The prepared samples of SiNWs and SiNWs:Au-NPs were immersed in aqueous solutions of MB or RB with molecule concentrations varying from 10^−15^ to 10^−6^ M for 20 min to implement the molecule adsorption. Then, the samples were dried in air at room temperature.

### 3.5. Measurement Techniques

The morphology and size of SiNWs were investigated by using a scanning electron microscope (SEM), Zeiss Crossbeam 540. The same SEM apparatus was used to measure the size of deposited Au-NPs on the surface of c-Si wafers. The size distribution of plasmonic NPs was obtained from their SEM images using the image processing software ImageJ. The porosity of SiNW arrays was estimated from the corresponding top-view SEM images using the box-counting method (Supplemental Figure S1). Samples of SiNWs and SiNWs:Au-NPs were also examined using a transmission electron microscope (JEOL JEM, 1400 Plus, Japan) operating at 200 kV. Spectra of the total (both diffused and specular) optical reflectance in the region from 400 to 1000 nm were measured with a Perkin Elmer Lambda 35 spectrometer (Perkin Elmer, USA). Raman scattering measurements were performed by using a Raman microscope (Ntegra Spectra, NT-MDT, Russia) with 600 grooves/mm grating. The laser beam of a HeNe laser (632.8 nm, 1 mW) was focused on the top of the sample surface, and the laser spot diameter was about 10 μm, while the integration time for each spectrum was 100 s. The characteristic Raman peak of c-Si wafer at 520.5 cm^−1^ was used to calibrate the spectrograph for possible fluctuations in the Raman system. To check the reproducibility of the SERS spectral signals of MB and RB adsorbed on the surfaces of SiNWs and SiNWs:Au-NPs, we carried out measurements at different sample regions, and almost identical intensities and positions of the Raman bands were obtained. The spectra were recorded 10 times and then averaged. All measurements were conducted in air at room temperature.

## 4. Conclusions

To summarize, we prepared the SERS substrates based on the MACE-grown SiNWs of controlled porosity followed by the deposition of plasmonic Au-NPs on the surfaces of SiNWs. The prepared samples were explored as SERS templates for the sensitive detection of adsorbed molecules of Methylene blue and Rhodamine B. Significantly, such dye molecules could be easily detected at an extremely low concentration of the order of 1 fM. Both the enhancement factor and minimal detectable concentration of analyte can obviously be improved by further optimizing the morphology of SiNWs and the concentration of deposited Au-NPs.

Our results demonstrate that SiNWs:Au-NPs structures with desired porosity possess the optimal conditions for the Raman scattering, which is combined with surface plasmon resonance, and that they substantially enhanced the SERS signal of adsorbed molecules. The operationally simple and inexpensive applied synthesis method yielding dense arrays of SiNWs with Au-NPs for the SERS detection of small target molecules can be useful in the preparation of SERS templates for biosensing applications.

## Figures and Tables

**Table 1:** Enhancement factor for selected Raman lines of MB (10^−6^ M) in samples of SiNWs:Au-NPs with different porosity, *P*. The assignment of the Raman lines is performed according to Ref. [47].

Frequency (cm^−1^)	Assignment	Enhancement Factor (10^4^)		
		*P* = 55%	*P* = 72%	*P* = 83%
450	α (C―N―C)_AMG_	5.33	3.10	0.86
500	α (C―N―C)_AMG_	2.92	1.45	0.50
595	α (C―N―C)_AMG_	1.52	0.89	0.32
669	α (C―C―C)_Ring_	1.01	0.45	0.21
769	ν (C―N)_AMG_ α (C―N―C)_Ring_	3.09	1.27	0.60
874	α (C―C―C)_Ring_	1.75	1.02	0.41
949	ρ (CH_2_); β (CH)	3.74	1.67	0.77
1035	β (CH); ν (C-S)	1.12	0.58	0.22
1181	ρ (CH_3_); β (CH)	2.06	0.78	0.34
1301	β (CH); ν (C―N)_Ring_	2.27	0.94	0.39
1392	ν(C_9_―N_10_); ν(C_3_―N_2_)	6.3	2.78	1.09
1444	α(N―C―H)_AMG_ ν(C―C)_Ring_/ν(C―C)_Ring_	16.68	7.96	2.89
1625	{ν(C―C)/ν(C―N)}_Ring_	6.13	2.58	1.09

## Data Availability

Not applicable.

## Supplementary Materials

The following are available online at https://www.mdpi.com/article/10.3390/ijms23052590/s1.
