# Supplementary material for: Surface-Enhanced Raman Scattering from Dye Molecules in Silicon Nanowire Structures Decorated by Gold Nanoparticles
Source: Int J Mol Sci. 2022 Feb 26;23(5):2590. doi: 10.3390/ijms23052590 (PMC8910339; doi:10.3390/ijms23052590)
Supplement: Supplementary file 1 [file ijms-23-02590-s001.zip › ijms-1581671-supplementary.pdf]

International Journal of *Molecular Sciences*

MDPI

Supplementary Materials

Surface-Enhanced Raman Scattering from Dye Molecules in Silicon Nanowire Structures Decorated by Gold Nanoparticles

Saltanat B. Ikramova [1], Zhandos N. Utegulov [2,*], Kadyrjan K. Dikhanbayev [1], Abduzhappar E. Gaipov [3], Renata R. Nemkayeva [4], Valery G. Yakunin [5], Vladimir P. Savinov [5] and Victor Yu Timoshenko [5,6,*]

[1] Faculty of Physics and Technology, Al-Farabi Kazakh National University, 71, Almaty 050040, Kazakhstan; ykramova.saltanat@kaznu.kz (S.B.I.); dksolar2017@gmail.com (K.K.D.)
[2] Department of Physics, School of Sciences and Humanities, Nazarbayev University, Nur-Sultan 010000, Kazakhstan
[3] Department of Medicine, Nazarbayev University School of Medicine, Nur-Sultan 010000, Kazakhstan; abduzhappar.gaipov@nu.edu.kz
[4] National Nanotechnology Laboratory Open Type, Faculty of Physics and Technology, Al-Farabi Kazakh National University, Almaty 050040, Kazakhstan; quasisensus@mail.ru
[5] Faculty of Physics, Lomonosov Moscow State University, 119991 Moscow, Russia; yvg51@bk.ru (V.G.Y.); savinov1983@yahoo.com (V.P.S.)
[6] Lebedev Physical Institute of the Russian Academy of Sciences, 119991 Moscow, Russia
* Correspondence: zhutegulov@nu.edu.kz (Z.N.U.); timoshen@physics.msu.ru (V.Y.T.)

Porosity estimation

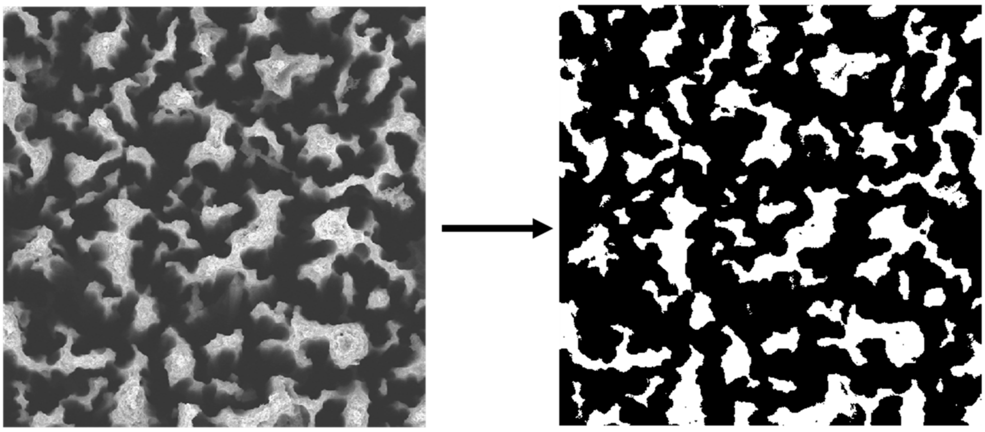

(a) **(b)**

Figure S1. Illustration of the box-count method to estimate the porosity of SiNWs arrays: **(a)** SEM top-view image and **(b)** the same image in the “black-white” format. Porosity of SiNWs arrays is estimated by using the following equation:

$$P = 1 - \frac{N_w}{N_w + N_b} \tag{S1}$$

where N_w and N_b are numbers of black and white pixels, respectively.

EDX analysis

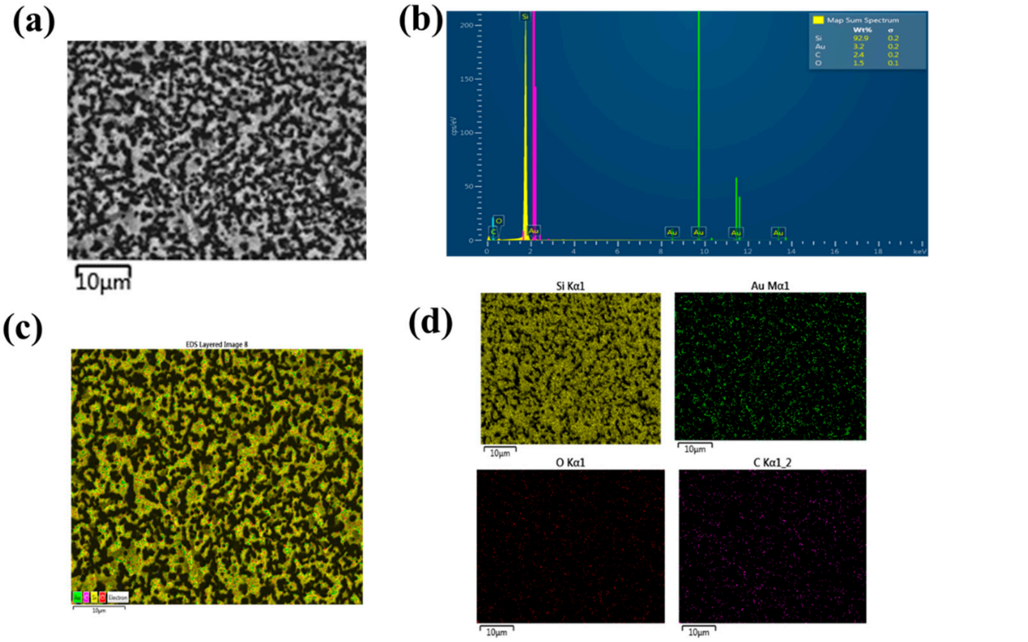

Figure S2. **(a)** SEM image of SiNWs:Au-NPs (P = 55%), **(b)** EDX spectrum of this sample, **(c)** merged elemental image and **(d)** images for selected elements (Si, Au, O, C).

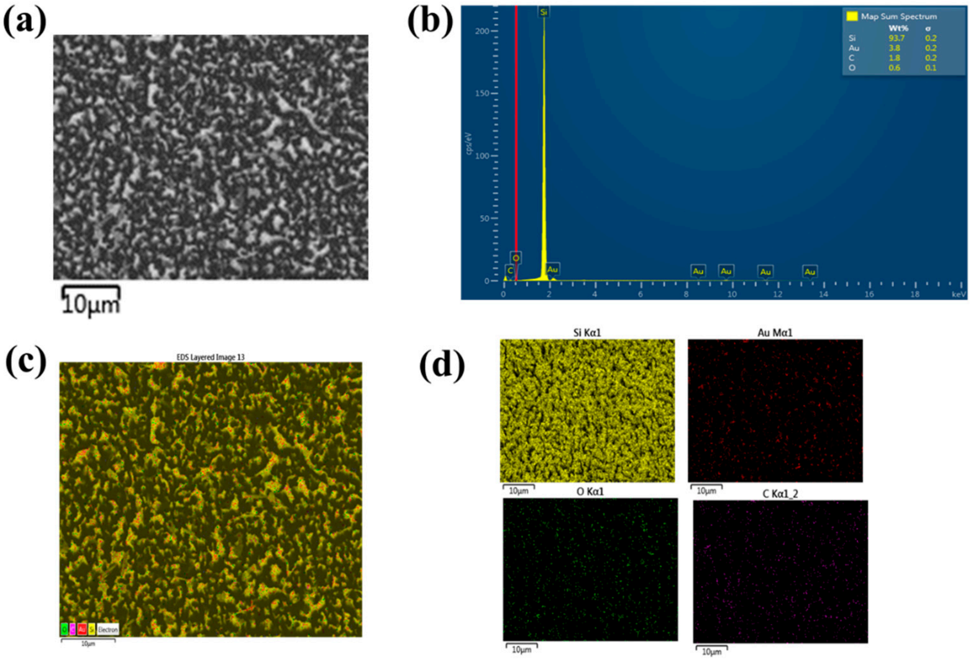

Figure S3. **(a)** SEM image of SiNWs:Au-NPs (P = 72%), **(b)** EDX spectrum of this sample, **(c)** merged elemental image and **(d)** images for selected elements (Si, Au, O, C).

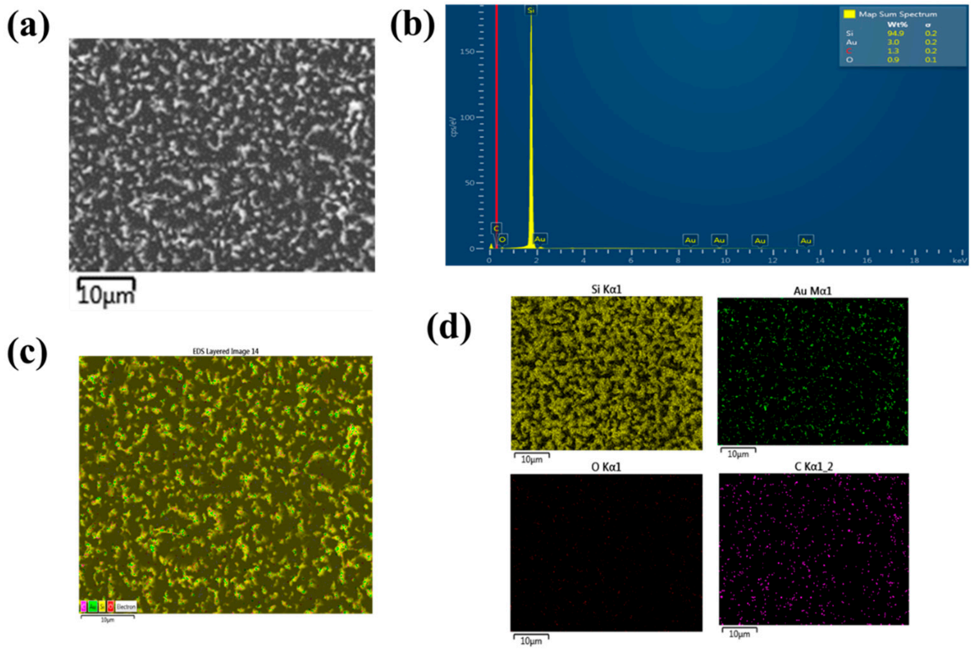

Figure S4. **(a)** SEM image of SiNWs:Au-NPs (*P* = 83%), **(b)** EDX spectrum of this sample, **(c)** merged elemental image and **(d)** images for selected elements (Si, Au, O, C).

Table S1. Summarized results of the elemental content of SiNWs:Au-NPs with different porosity.

Samples	Elements	Wt(%)	At (%)
P = 55%	Si	92.9	91.5
	Au	3.2	0.4
	C	2.4	5.5
	O	1.5	2.6
P = 72%	Si	93.7	94.0
	Au	3.8	0.5
	C	1.8	4.2
	O	0.7	1.3
P = 83%	Si	94.9	95.1
	Au	3.0	0.4
	C	1.2	2.8
	O	0.9	1.6

EF estimation

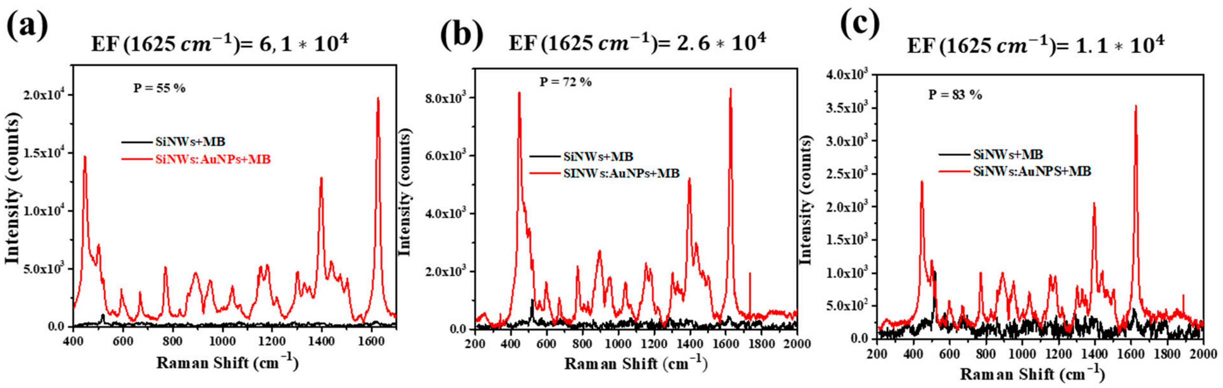

Figure S5. SERS spectra for SiNWs and SiNWs:Au-NPs with deposited MB (1 μM) for the samples with different porosity: **(a)** *P* = 55%, **(b)** *P* = 72%, and **(c)** *P* = 83%. The spectra are shown after subtraction of the photoluminescent background.

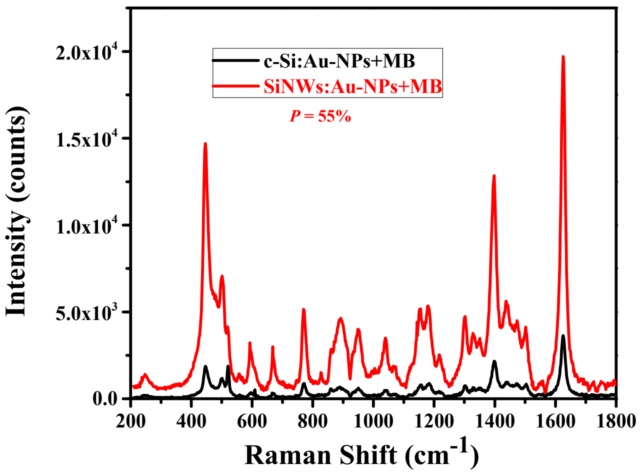

Figure S6. SERS spectra of MB (1 μM) deposited on c-Si:Au-NPs (black line) and SiNWs:Au-NPs with *P* = 55% (red line). The spectra are shown after subtraction of the photoluminescent background.

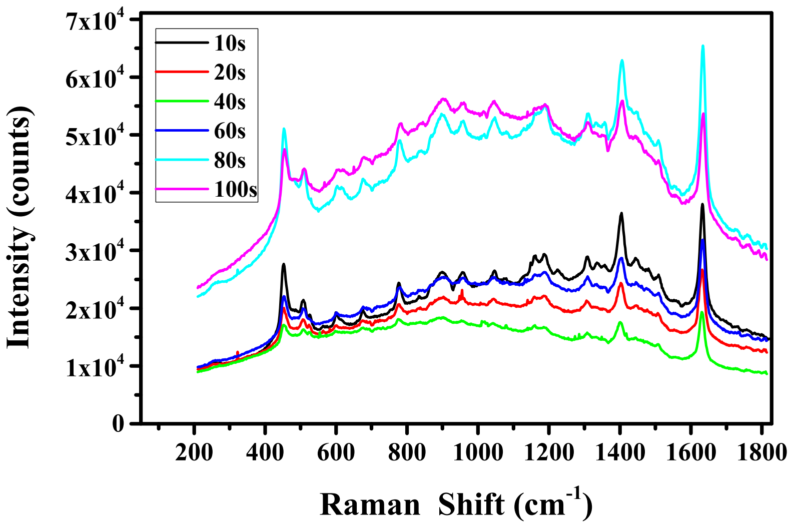

Figure S7. Spectra of the Raman scattering and photoluminescence of MB (1 μM) for samples of SiNWs:Au-NPs with *P* = 55% and different time of Au-NP deposition varied from 10 to 100 s.
